# Interpersonal Psychotherapy’s problem areas as an organizing framework to understand depression and sexual and reproductive health needs of Kenyan pregnant and parenting adolescents: a qualitative study

**DOI:** 10.1186/s12884-022-05193-x

**Published:** 2022-12-15

**Authors:** Manasi Kumar, Obadia Yator, Vincent Nyongesa, Martha Kagoya, Shillah Mwaniga, Joseph Kathono, Isaiah Gitonga, Nancy Grote, Helena Verdeli, Keng Yen Huang, Mary McKay, Holly A. Swartz

**Affiliations:** 1 Department of Psychiatry, University of Nairobi, 00100 (47074), Nairobi, Kenya; 2 Brain and Mind Institute, Aga Khan University, Nairobi, Kenya; 3 Nairobi Metropolitan Services, Nairobi, Kenya; 4 Vrije University, Amsterdam, Netherlands; 5 Department of Psychiatry, University of Nairobi, Kenya and Nairobi Metropolitan Services, Nairobi, Kenya; 6 Department of Psychology, National University of Ireland, Maynooth, Ireland; 7 School of Social Work, University of Washington, Seattle, USA; 8 Global Mental Health Lab, Columbia University, New York, USA; 9 New York University, New York, USA; 10 Vice Provost of Interdisciplinary Initiatives, University of Washington, St Louis, USA; 11 Department of Psychiatry, University of Pittsburgh, Pittsburg, USA

**Keywords:** Interpersonal problems, Mental health, Pregnant adolescents, Poor social support, Stress, Depression, Kenya

## Abstract

**Background:**

Peripartum adolescents experience significant interpersonal transitions in their lives. Depression and emotional distress are often exacerbated by adolescents’ responses to these interpersonal changes. Improved understanding of pregnancy-related social changes and maladaptive responses to these shifts may inform novel approaches to addressing the mental health needs of adolescents during the perinatal period. The paper aims to understand the sources of psychological distress in peripartum adolescents and map these to Interpersonal Psychotherapy’s (IPT) problem areas as a framework to understand depression.

**Method:**

We conducted interviews in two Nairobi primary care clinics with peripartum adolescents ages 16–18 years (*n* = 23) with experiences of depression, keeping interpersonal psychotherapy framework of problem areas in mind. We explored the nature of their distress, triggers, antecedents of distress associated with an unplanned pregnancy, quality of their relationships with their partner, parents, and other family members, perceived needs, and sources of support.

**Results:**

We found that the interpersonal psychotherapy (IPT) framework of interpersonal problems covering grief and loss, role transitions, interpersonal disputes, and social isolation was instrumental in conceptualizing adolescent depression, anxiety, and stress in the perinatal period.

**Conclusion:**

Our interviews deepened understanding of peripartum adolescent mental health focusing on four IPT problem areas. The interpersonal framework yields meaningful information about adolescent depression and could help in identifying strategies for addressing their distress.

## Background

Pregnant adolescent girls are more vulnerable than pregnant adult women due to limited social networks, lack of financial independence, and underdeveloped physical and cognitive functions [1]. When pregnancy among school girls is seen as promiscuity, a consequence of poor parenting from their mothers, and shameful for their immediate family and entire family lineage, socio-cultural beliefs exacerbate the stress of early unanticipated pregnancy [2]. Research shows that pregnant adolescents experience several negative life challenges due to the pregnancy, including lack of emotional, and social support, poor health care access, social stigma, and problems associated with impending transition to adolescent motherhood [3]. It has further been suggested that well-documented adverse personal and community experiences during childhood contribute significantly to pregnant youth's social problems [4]. Many pregnant adolescents are unlikely to complete their education, making them less qualified for rewarding jobs [5]. Adolescent pregnancy constitutes a risk factor for maternal mortality [6–8]. These stressors likely contribute to emotional distress and negatively affect their long-term well-being.

Adolescent pregnancy rates (10–19 years) within Sub-Saharan Africa are among the world's highest [9]. The Global Childhood report published by Save the Children humanitarian organization estimates Kenya's teenage birth rate in 2016 at 82 births per 1,000 girls for age 15–19; and one in eight (12%) Kenyan girls aged 15–19 got married between 2013 to 2018 [10]. Approximately 18% of the country’s teenage girls (15–19 years) have begun childbearing or are already mothers, and about 13,000 drop out of school every year due to early unanticipated pregnancy [11]. In studies carried out earlier in Kenya, 32.6% of 176 pregnant adolescents aged 15–18 years were found to have significant depressive symptoms using the Patient Health Questionnaire -9 cut-off of 15 [12]. Individual predictors of depressive symptoms were: absence of social support, being younger, experience of a stressful event, and living with HIV/AIDS. We also know that half of all mental health conditions start at 14 years of age, but most cases are undetected and untreated [13]. Multiple levels of mental health problems are documented in adolescents in Sub-Saharan Africa. One in 7 children and adolescents has significant emotional difficulties, with 1 in 10 (9.5%) having a specific psychiatric disorder [14]. A recent Global Burden of Disease (GBD) study noted that the global coverage of prevalence data for mental disorders in children and adolescents is limited. Many low-and middle-income countries (LMICs) were poorly represented in the available prevalence data. For example, at a population level no region in Sub-Saharan Africa had more than 2% coverage for any psychiatric disorder for children and adolescents [15].

### Revisiting Interpersonal theory to situate the problem of adolescent mental health in LMICs

Harry Stack Sullivan’s theory of personality development was based on the belief that individuals’ interactions with others, especially significant others, determine their sense of security, sense of self, and the dynamism that motivates their behavior. The dynamisms that are distinctively human characterize one's interpersonal relations and function primarily to satisfy some basic needs of the individual [16]. Interpersonal theory's emphasis on the "interpersonal situation" includes both proximal description of overt behavioral transactions and covert psychological processes that mediate interaction, including the formation and function of internalized mental representations of self, others, and social structures and roles. Interpersonal theory’s unique contributions to the understanding of human psychology include well‐articulated structural models to describe interpersonal behavior and the patterned regularity of reciprocal human transactions that spur interpersonal behaviors [17]. Weissman and Klerman, amongst other Interpersonal Psychotherapy (IPT) researchers, have underscored the need to understand individuals’ interpersonal relationships and social roles to identify triggers of depressive symptoms [18]. We are now using the IPT framework in Kenya to understand triggers for poor mental health and relationship distress in pregnant and parenting adolescents in Kenya. In addition, the framework offers an opportunity to study relationship context in diverse cultures, especially in Sub-Saharan Africa, where rapid globalization alters living conditions, socialization, traditional practices, and psychosocial well-being of families and young people.

The transition to adolescence represents a critical developmental period associated with increased vulnerability to depressive symptoms [19–21]. This is especially true among Sub-Saharan adolescent girls who face additional challenges within their families and social environments, including financial problems, low sexual and reproductive health literacy, and culturally related female gender discrimination.

### Application of Interpersonal psychotherapy and four problem areas in the context of peripartum adolescent mental health

#### Interpersonal problem areas

Depression in pregnant adolescents is tied to interpersonal problems. In the IPT framework, these are categorized as *Grief and loss*: Grief occurs when a loved one dies; in the event of the death of a loved one or parents, most adolescents start taking care of younger siblings, move to live with other family members, quit school due to lack of school fees, or move to urban areas in search of employment. This is when they are most vulnerable to predatory relationships, such as men who mislead them with temporary emotional or financial support, which disappears when the adolescent becomes pregnant. In some instances, the adolescent moves to stay with the man who convinces her to stop working, resulting in significant financial and emotional challenges when the man abandons her. These all too common scenarios are often associated with elevated depressive symptoms.

#### Role Transitions/life changes

Pregnancy, by definition, is a life changing event. It requires one to prepare for childbearing and the needs of a newborn. Commonly, adolescents struggle to find income-generating activities, leading to emotional and financial insecurity. For adolescents, the added burden of abruptly transitioning from a school-going youth to unanticipated motherhood is associated with loss of chumships, social support, and normative social roles. These additional challenges associated with the pregnancy-related role transition increase vulnerability for poor mental health.

#### Interpersonal conflicts/role disputes

Adolescent pregnancies, in most cases, lead to conflicts between the adolescent and her family members. An adolescent who becomes pregnant while in school might face ridicule from classmates and peers and mistreatment from teachers. Not uncommonly, she may drop out of school, which lead to further disagreements with her parents or core family members. When the person responsible for the pregnancy denies it, it creates a lot of tension in the household since there is a lack of support for the adolescent across her social networks, which is associated with low mood.

#### Interpersonal deficits (loneliness/social isolation)

Interpersonal deficits refers to longstanding impoverished or contentious relationships. In traditional IPT, the interpersonal deficits area is used for individuals who have difficulty starting or maintaining relationships. In this instance, we expand the deficits definition to encompass the experience of social isolation endured by pregnant adolescents as a result of challenging life circumstances, hostile domestic environment with disagreements with family members and peers, she decides to be on her own, avoids any kind of company, and the shame that comes with the pregnancy also makes her more isolated. In such a scenario, she deals with her problems without seeking support from anyone and makes her own decisions, good or bad, and in most cases, depression sets in, emanating from isolation and absence of supportive relationships. The additional adverse impact of social isolation during this formative developmental stage is that it may lead to developmental gaps in prosocial behavior and the formation of intimate relationships.

### Sociocultural challenges facing pregnant adolescents in Sub-Saharan Africa

The specific challenges of evolving adolescent relationships and early pregnancy in a developing country are manifold. Peri-urban settlements in Nairobi provide a backdrop for the current study and the lives of the youth interviewed here. The social context of these settlements is complex. On the one hand, they provide many good social support and networking opportunities through extended families and close communities with shared language, religions, and values and a sense of camaraderie where people will look out for one another; on the other hand, there is rampant violence, poor sanitation, and limited access to health and civic services [22]. In addition, predatory behaviors by men are commonly documented, given high rates of unemployment, informal labor, and substance misuse [23, 24]. Adolescent girls and women are particularly vulnerable to predation. Interpersonal relationships within the families are marked by protective mechanisms to safeguard oneself, and one’s outside relationships with peers. Neighbors and close community members act as sources of support. However, pregnancy threatens adolescent girls’ access to these protective mechanisms.

The current study is part of a pilot implementation trial that tests culturally adapted group interpersonal psychotherapy (IPT-G) for pregnant adolescents seen in Nairobi primary maternal and child health clinics. This paper aims to understand interpersonal challenges facing peripartum adolescents experiencing unplanned pregnancies. The specific goal here was to carry out in-depth interviews with adolescents to understand their sources of distress better and bolster the intervention with additional implementation strategies to improve adolescent mental health outcomes and increase uptake of evidence-based interventions.

## Methods

### Participants

We Interviewed 23 pregnant and parenting participants using a semi-structured questionnaire addressing various mental, psychosocial, and interpersonal challenges associated with pregnancy and motherhood. Participants were not taking part in treatment, since these were key informant interviews to help identify aspects of IPT, applicable to pregnant and parenting adolescents and carry out necessary modifications in the intervention and implementation strategy for the study. The participants were 16 to 18 years old (mean age was 17.7 years (SD—0.6)). Twelve participants were married, and eleven were living with their caregivers. Most participants were Christian; nine participants reported that both parents raised them, thirteen reported being raised by one parent due to separation, while one grew up as an orphaned child. The mean age during the first sexual experience was 16 years (the first sexual encounter ranged from 13 to 18 years). Eight of the participants had completed secondary education; four completed primary education only. Eight started but never completed secondary education, while two had less than primary education. One participant had some college education (see Table 1).

**Table 1:** Sociodemographic information

	**Category**	**Frequency**	**Percentage (%)**	**Mean & standard deviation**
Age (Years)	16–17	5	21.7	17.7 (SD 0.6)
	18	18	78.3	
Education	Primary (Not completed)	2	8.7	
	Primary (Completed)	4	17.4	
	Secondary (Not completed)	8	34.8	
	Secondary (Completed)	8	34.8	
	College	1	4.3	
Marital status	Single	11	47.8	
	Married	12	52.2	
Gestation	Pregnant	11	47.8	
	Delivered	12	52.2	
Age of first sexual experience (Years)	13–15	5	21.7	16 (SD 1.4)
	16–18	18	78.3	
Marital status of parents	Married	11	47.8	
	Separated	12	52.2	
EPDS depression	None or minimal depression	7	30.4	11.1 (SD 7.0)
	Mild depression	7	30.4	
	Moderate depression	6	26.1	
	Severe depression	3	13.1	

This table shows characteristics of adolescents who participated in our interviews

The average interview duration was 40 minutes. We conducted interviews with adolescents in May- June 2019, and out of 23 adolescents, 70% experienced elevated depressive symptoms (mean score of 11.1 (SD 7.0) as assessed on the Edinburgh Postnatal Depressive Scale (EPDS), about 39% of our participants were in the moderate to severe depressive symptoms range and 13% in the severe depressive symptoms range.

## Setting description and location

### Kariobangi North and Kangemi Health Centers

Kariobangi health center is a level three facility under the Nairobi Metropolitan Services, formerly the Nairobi County Council. A level three facility includes health centers, maternity homes, and sub-district hospitals. It is located in a low-income residential area in the northeastern part of Nairobi, Kenya. Kariobangi consists of lower middle class areas and slum-type regions (Kariobangi north) with 18,903 residents [25]. Kangemi Health Center is also a level three facility under the Nairobi Metropolitan Services. It is located in a slum in Nairobi City within a small valley on the city’s outskirts. The 2019 Kenya population and housing census show that Kangemi had 116,710 residents [25]. Kangemi runs a 24/7 maternity service and thus attracts many young women for these services.

### Ethical approval and clearances

The study was approved by the Kenyatta National Hospital/University of Nairobi ethical review committee (approval no. P694/09/2018). In addition, approval was received from Nairobi County Health no. CMO/NRB/OPR/VOL1/2019/04, and a permit from Kenyan National Commission for Science, Technology, and Innovation (NACOSTI/P/19/77705/28063) was obtained.

### Data collection and analysis

Participants were recruited through engagement with the health facility In-charges, and community health volunteers (CHV) were recruited to identify eligible participants. Each CHV works under a community health assistant who oversees their activity. The eligible participants provided informed consent under the supervision of two authors in the health facility. The facility provided a private space for interviews. Participants were offered transport reimbursement and snacks upon completion of the interview.

The interviews were conducted in both Kiswahili and English and audio-recorded. One of the authors then transcribed these in English, and other authors read the transcripts.

Data were analyzed using a two-stage process- manual coding by the authors and later through triangulation via NVivo [26] by a data analyst. The coding was meant to be inductive and deductive, aimed at expanding on IPT problem areas and deepening the experiences of adolescents and their depression-associated distress and life circumstances. It was inductive around collating common experiences around four problem areas and capturing idioms of distress around unplanned pregnancy and motherhood by reviewing the transcripts individually, and it was deductive with regards to mapping problems along the framework we have extended here using NVivo. Our deductive analysis process was to develop emerging themes that further articulate this framework (see Table 2). We carried out thematic analysis- reviewing the themes, narrative vignettes and collating ideas around the formal coding via NVivo.

**Table 2:** Interview guide and methodological framework

Interview domains
***I Interview guide for depressed adolescent***
1.Biographical history
• Kindly tell us about your family background • What level of education did you attain (Reason) • Do you have a partner? • Do you live with him or with your parents? Do you live with someone else?
2. Circumstances leading to pregnancy
• At what age did you have your first sexual experience • Please elaborate on the circumstances that led to that experience • Would you tell us about your personal experience with pregnancy • What was the reaction of your boyfriend/ partner (s) and immediate family when you announced your pregnancy •Would you tell us how your neighbors, relatives and friends treated you after learning about your pregnancy
3. Challenges faced with the pregnancy
• Economic challenges (Food, clothing, shelter, access to medical services • Social challenges (Social support, level of education, domestic violence, sexual abuse and alcohol/substance abuse)/ • Medical challenges (STI/HIV, mental illnesses)/ • Antenatal Depression • What is your understanding of the term depression • What is your experience with depression • What can girls in your situation do to cope with depression
***II Interpersonal psychotherapy conceptualization of interpersonal problem areas:***
The problem areas are IPT key components highlighting the problems triggering depression (Grief/loss, Interpersonal disputes, Role transitions, and Social isolation) **Grief/loss** – this problem area is used for the experience of losing a loved one to death **Interpersonal disputes**-Is if there is a misunderstanding between two parties given the needs/requirements **Role transitions**- If there are changes in one's life, for example, being a child to becoming an adolescent **Social isolation**-social deficits and inability to make or maintain relationships

A box showing the interview guide questions we used during key informant interviews

The thematic analysis pointed to the need to address different facets of relationships and socioeconomic and structural challenges contributing to depression and mental distress in this population. We used the IPT interpersonal problem areas as a framework to contextualize depression in this sample, as shown in Table 2. As the authors have previously carried out qualitative work with the same population [1, 3], this analysis aimed to provide a more detailed focus on depression and the associated experiences of psychological, interpersonal, and social distress.

## Results

**Fig. 1:**
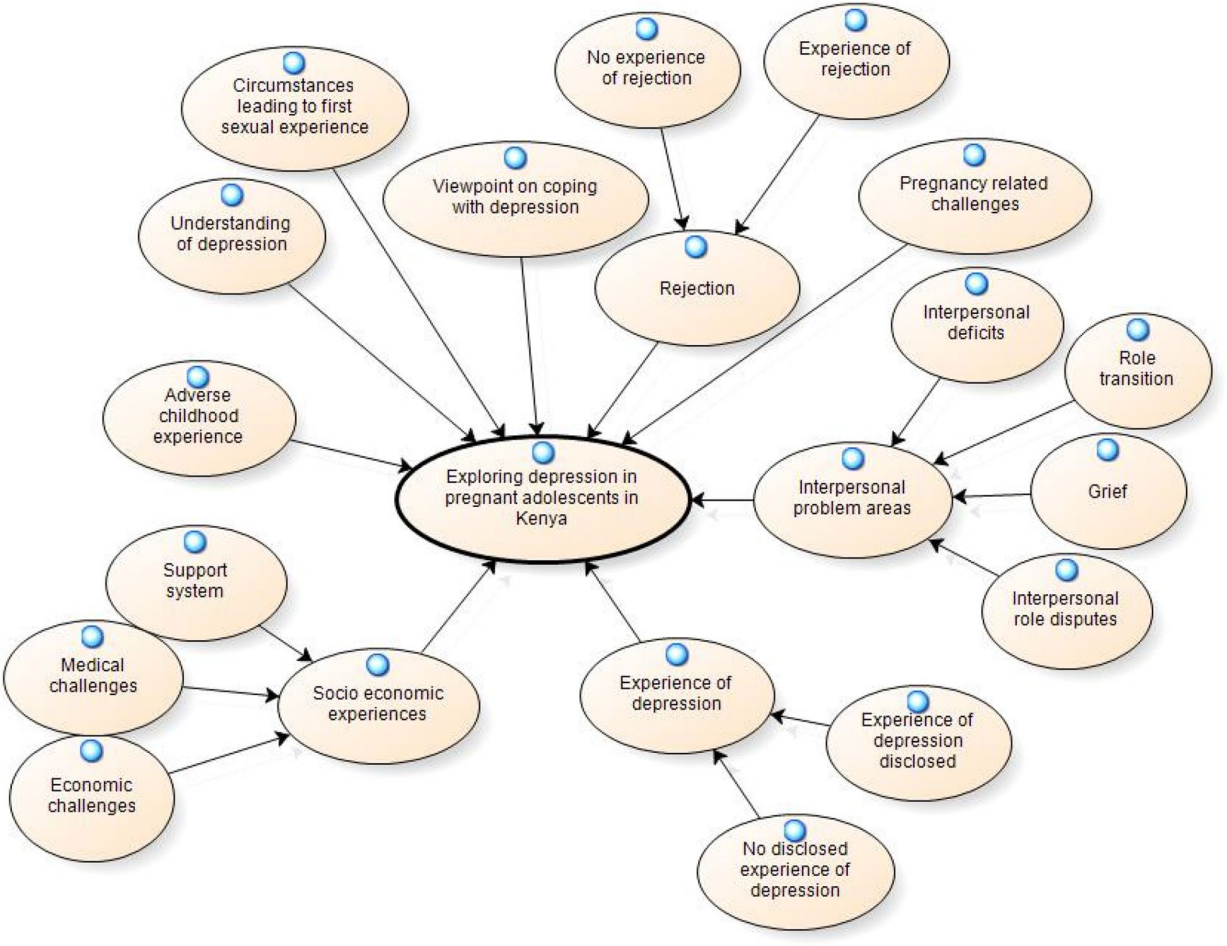
Intersecting themes around depression

We generated a range of themes from a conceptual framework depicting interpersonal problems and participant interviews. All the participants were encouraged to share their experiences through follow-up questions during the interview sessions. All four IPT problem areas were present in the sample, and interpersonal relationships were identified as a significant source of concern to our adolescent participants as much of adolescent pregnancy and motherhood experiences revolve around a challenging and conflictual relationship experience (see Table 3 and Fig. 1).

**Table 3:** Interview themes with corresponding relevant quotations

Overall theme	Relevant quotations from participants	Sub-themes
**Pregnancy, Adverse Childhood Experience and Rejection from family and relatives**		
Pregnancy related health difficulties	*I was stressed [some silence] because I used to wake up in the morning feeling tired, vomiting* (AD07KG, 18 years) *Yes, headache, fever, I was feeling cold throughout *(AD07KG, 18 years) *Vomiting, laziness; you don't feel like doing anything, getting tired quickly whenever you do something* (AD11KG, 18 years)	Morning sickness, tiredness, fever and headache “Pregnancy didn’t like him (partner)”…. Myth?
Adverse Childhood experiences	*The process of carrying the pregnancy was just okay [Inaudible] it used to reach a time then he just gets angry so I could try to talk to him but he is just angry and I wondered what it was; I thought of getting out of his house but I thought it was not a good thing; I told my sister from my other mother is the one who came to help me. She told me that my pregnancy didn't like him but when I give birth things would be fine, that is when I became fine* (AD09KG,17 years)	Growing up in a polygamous family with limited resource
Experiences of rejection	*My father has two wives and my mother is the second wife; now the first wife's children were studying in boarding schools and he was paying their whole amount of school fees but for me I was in a day school and I was being sent away from school so it affected me so much* (AD13KG, 18 years) *Then for example farming we used to do together but whenever we harvest, all the food used to go to the first wife* (AD13KG, 18 years) *Currently it is just that you know when mum gets angry, she never talks to you* (AD11KG, 18 years) *He got angered and even denied that he is not responsible for the pregnancy* (AD08KG, 18 years) *He started saying that he was not responsible. I just told him if it belongs to someone else then it is okay* (AD01KG, 18 years) *They asked me to abort it or else they would beat me up *(AD01KG, 18 years) *They never accepted it at first* (AD05KG, 18 years)	Rejection from mother; the father of unborn child denies responsibility Family members rejecting the unborn baby and wanting abortion
**Experience and understanding of depression**		
Experiences of depression	*It is too much thinking* (AD02KG, 18 years) *Depression is like stress, right? Thoughts, thinking too much *(AD07KG, 18 years) *Depression is being stressed so much until you stay at the same place; kind of you feel that there is a hole at that same place *(AD11KG, 18 years) *Being stressed* (AD08KG, 18 years) *It is being shocked* (AD03KG, 18 years) *Somebody who is stressed* (AD13KG, 18 years) *Depression [Silence] depression is like when you are alone, and nobody is supporting you *(AD12KG, 18 years) *I have no idea* (AD01KG, 18 years) *This is when one is so much stressed to a point of having negative impacts* (AD05KG, 18 years) *It is too much thinking* (AD02KG, 18 years)	Summarized it as feeling of anger, quarrelling and feeling stressed up
Understanding of depression	*Depression is like stress, right? Thoughts, thinking too much* (AD07KG, 18 years) *It is being shocked* (AD03KG, 18 years) *Somebody who is stressed* (AD13KG, 18 years) *Depression [Silence] depression is like when you are alone, and nobody is supporting you* (AD12KG, 18 years) *I have no idea*. (AD01KG, 18 years) *This is when one is so much stressed to a point of having negative impacts* (AD05KG, 18 years)	Two statements were commonly used to describe depression; 1. Thinking too much 2. Being stressed Also lack of support while in distress was associated with depression
Denying experience of depression associated with lack of knowledge or willingness to engage	*It is not there *(AD02KG, 18 years) *No, I haven't gone through [depression]* (AD07KG, 18 years) *I have never; maybe blood pressure* (AD11KG, 18 years) *No* (AD12KG, 18 years)	They may not have depression or there is a knowledge gap (they do not understand depression) or not well probed
**Interpersonal problem areas**		
Grief	*Yes; mum passed on* (AD08KG, 18 years) *You know we were raised by uncle; mum's brother. Mum died in two thousand and four* (AD13KG, 18 years)	Death of a parent and being raised by a relative/guardian
Role transition	*Obviously, I was not expecting (the pregnancy)* (AD02KG, 18 years) Pregnant while still in school (AD08KG, 18 years) *I cannot go back home while I am pregnant* (AD03KG, 18 years) *I used to go to school while pregnant but when my mother noticed that, I decided to move out* (AD01KG, 18 years) *the issues of girls not paying attention to the parents, running around with boys, so my sister took me and brought me here so that I study from here; I came here in the same twenty seventeen then I again went to live with another sister of mine that is when I became pregnant *(AD09KG, 17 years)	Role transition was experienced especially by those who were in school transitioning to early motherhood. (Others probably were already in marriage or not in school and therefore it did not come out
Interpersonal role disputes	*He was worried because my parents could come anytime to pick me up. it was just the parents being frustrated they quarreled because I had not completed my studies *(AD07KG, 18 years) *He said that I should abort to show that the child is his, so I decided it is better that I forget him* (AD11KG, 18 years) c*ontinued…………It was not that easy because there are things that we went through; sometimes mum doesn't talk to you because she is also disappointed* (AD11KG, 18 years) *They became angry because they never thought that I was in form four, they thought that I am in form two; so upon realizing that I was in form four they got angry* (AD08KG, 18 years) *They wanted me to go back home so that they can take me for a course, but I refused* (AD03KG, 18 years)	This was a dominant theme in the 4 PT problem areas – Parents/guardians disagreed and disappointed with the decision of the pregnant adolescent. This caused tension and frustration
Interpersonal deficits	*I don't usually have friends within* (AD11KG, 18 years) *As in I did not want to see anybody I just wanted to stay alone* (AD13KG, 18 years)	Experienced difficulty in forming relationships
Circumstances leading to first sexual experience	*No; I was obviously seduced* (AD02KG, 18 years) *He didn't give me alcohol, he just talked to me* (AD07KG, 18 years) *It is just wanting because even that relationship started as a result of peer pressure; you want to be seen* (AD11KG, 18 years) *It is just the way you hear that others have done it and you also want to try it *(AD08KG, 18 years) *You just know life sometimes you don't have pads, you don't have whatever - books, whatever; so you get somebody who tells you "I will buy for you," that way* (AD13KG, 18 years) *It was just because of peer pressure* (AD12KG, 18 years) *That boy convinced me, and I couldn't resist because I was in love with him* (AD09KG, 18 years)	Peer pressure was identified as a cause for first sexual experience Also lack of basic necessities; books, pads (sanitary towels)
**Coping with depression**		
Viewpoint on coping with depression	*They just have to accept themselves* (AD02KG, 18 years) *Just stop thinking and give birth rather than aborting because aborting itself is a problem and you will be adding stress to yourself* (AD07KG, 18 years) *You just try to share whatever is in your mind with your closest person …………..Or you try to seek advice from maybe a certain counselor* (AD11KG, 18 years) *Making them to form groups where they can help each other* (AD08KG, 18 years) *They should pray so much to God to help them* (AD11KG, 18 years) *I can say that if there is a person who wants to help them can assist them because most girls we find ourselves in certain problems because we don't have the potential I mean you don't have somebody to assist you (economically)* (AD13KG, 18 years) **continued**: *If they have somebody who can sit with them to have stories or if they have something to do. You know the more you stay alone is when you start to think of several things, yeah;* (AD13KG, 18 years)	Several viewpoints came up: Accepting and moving on/avoid abortion Religion/prayer Talking to close friend Seeking professional counselor Group support Livelihood support
**Socio economic experiences**		
Economic challenges	*It is a challenge, but we are just trying; if I get out and get something *(AD11KG, 18 years) *I just used to feed on what they ate ……………in jobs I am told that I must have a certificate so as to qualify* (AD08KG, 18 years) *My uncle is the one who was paying for me but he reached a point where he got overwhelmed* (AD13KG, 18 years) *Sometimes we lack money until he is forced to take food on credit from some shops* (AD01KG, 18 years)	Unable to afford balanced meals Inability to secure job after dropping out of school
Medical challenges	*HIV…………Yes, I told him that "I am positive so I don't know if we will break," but he told me, "no, we will just go on"* (AD02KG, 18 years) *When I went for ANC clinic, I was told to go for ANC profile, and it was one thousand five hundred, yet I did not have money* (AD08KG, 18 years) *Like when I fall sick sometimes there is lack of money* (AD03KG, 18 years) *HIV* (AD01KG, 18 years)	Respondents highlighted HIV and also lack of money to pay for clinic as some of the challenges
**Support system and social support**		
Support system	*I stay with my mother *(AD02KG, 18 years) *My mother told me not to worry that is how life is, so I just accepted* (AD07KG, 18 years) *Yes. So when I came out of the clinic I told him that, "they have confirmed I am pregnant" [some silence] I told him, "they have confirmed I am pregnant," then he was like, "are you serious?" I told him, "it is true, here is the report written to me, and even the doctor will tell you," he said, "it is alright* (AD07KG, 18 years) *just the other insurance *(AD11KG, 18 years) *I went through a lot, I had difficult time, because by that time we had already separated with the father of the child so I was wondering about who would help me; but I was helped by my brother though I was just staying in the house, I was not going to school *(AD08KG, 18 years) *He said, "if you are pregnant then let us get married," and that is how it happened* (AD03KG, 18 years) *I just told him that, "I am feeling weird, I don't want food," then he told me that, "we will go to hospital," so after that he got happy* (AD12KG, 18 years) *Mum's NHIF then for the child I had Linda Mama *(AD12KG, 18 years) *He was happy, in fact he told me that he was responsible for the pregnancy, it made him feel happy* (AD09KG, 17 years)	Family support especially mother Support from partner/boyfriend NHIF
Support and acceptance	*Just good; they are okay with that* (AD02KG, 18 years) *They were happy; I even gave birth while at my parents' home*. (AD07KG, 18 years) *He said, "if you are pregnant then let us get married," and that is how it happened *(AD03KG, 18 years) *They treated me well* (AD13KG, 18 years) *He got happy then took me to their home and introduced me to his parents* (AD12KG, 18 years)	Acceptance and offering social and emotional support

Table showing themes, sub-themes, and quotes from our key informant interview participants

We report Interpersonal Psychotherapy’s problem areas by looking at the possible life events depicting losses within their family set-up that could predispose perinatal adolescents to vulnerability (*loss and grief*), unpleasant life events during unintended and unplanned pregnancy, and the new status of being a young mother (*role transition*), some limitations in communication associated with emotional and social relationships (*interpersonal deficits*), and challenges emanating from interpersonal deficits causing miscommunication and quarrel with family members or male partners (*role conflict*). The unpleasant life events affecting pregnant and parenting adolescents are then linked with mood changes and vice versa to understand how the participants find themselves depressed. The narrations of their life experiences by the participants in relation to severity of depressive symptoms as measured using EPDS validates our findings.

Table 3 highlights various themes drawn from our interviews. Figure 1 outlines the key themes reflected in Table 3, showing the interrelationships among these thematic areas. Again, the figure here maps experiential and qualitative associations and does not suggest causal mechanisms or imply any quantitatively assessed or derived associations.

### Risk factors for perinatal depression

Our participants narrated challenges within their interpersonal relationships emanating from either unintended, unplanned, or early pregnancies. Our study found that adverse childhood experiences (ACEs) preceded adolescent pregnancy. Some of the ACEs we identified were: growing up in a polygamous family with meager resources, lack of access to necessities, including food but also books, hygiene and health needs (such as undergarments, and sanitary towels), and in some instances, the death of a parent and being raised by a distant relative/guardian. The pregnancy is coupled with limited access to services, including mental health services, little family or community support, poor access to educational or vocational, parenting training, and often all of these occur without male partner support. For our study population, the news that one is pregnant causes much distress. Our participants recounted this was due to experiences of perceived rejection by the family, dropping out of school, being ridiculed by their peers, and male partners disowning responsibility for the pregnancy*.* In addition, the participants experienced life events affecting their interpersonal relationships, including grief and loss, an abrupt transition to young motherhood, conflicts with parents and male partner, and a sense of loneliness upon rejection by family and male partner (see Table 3). Several of our participants were screened for depression using self-reported EPDS tool, and these adverse experiences may have led to psychological disturbances, including hopelessness, low mood, high stress and anxiety, and low self-esteem and self-efficacy.

### Socioeconomic challenges and poor support structures

Participants in our current study were from poor urban settlements characterized by high poverty levels and barriers to accessing services and goods for basic needs. These factors predispose them to mental health problems and psychosocial adversities [3, 12, 27]. The vignettes below highlight these challenges.

“*Sometimes we lack money until he is forced to take food on credit from some shops.”*

*“It is a challenge, but we are just trying; if I go out and get something for food.”*

*“I just used to feed on what they ate ……………in jobs I am told that I must have a certificate to qualify.”*

*“My uncle is the one who was paying for me but he reached a point where he got overwhelmed.”*

In tandem with interpersonal psychotherapy framework of problem areas, we noted presence of interpersonal deficits where some participants experienced difficulties in forming relationships which aggravated their sense of loneliness.

*“I don't usually have friends within the community.”*

*“(As in) I did not want to see anybody ……I just wanted to stay alone.”*

The social isolation limited their capacity to rely on others for practical help and suggestions.

### Pregnancy, Adverse Childhood Experience, and Rejection from family and relatives

Pregnancy-related health problems and lack of appropriate education about their management were reported as a source of distress. One participant explained:

“*I was stressed because I used to wake up in the morning feeling tired, vomiting ………Yes, headache, fever, I was feeling cold throughout.”*

Participants reported adverse childhood experiences, including different forms of family dysfunction linked to polygamous marriages with limited and unequally shared resources and single (mother) parenting. However, these were not always directly linked to depressive symptoms during pregnancy, as articulated by them during the interviews. We did get a sense that our participants lived through challenging circumstances that created difficult choices before them, with limited support and ability to understand the psychological strain of these difficult circumstances.

“*My father has two wives, and my mother is the second wife; now the first wife's children were studying in boarding schools, and he was paying their whole amount of school fees but for me I was in a day school and I was being sent away from school, so it affected me so much ……Then for example farming we used to do together but whenever we harvest, all the food used to go to the first wife.”*

*“We are just two, my parents broke up …. I was still in kindergarten…Dad is not available.”*

Some participants reported interpersonal conflict following rejection from family and friends due to pregnancy. In some cases, the boyfriends denied responsibility for the pregnancy, while those who accepted suggested termination of the pregnancy.

*“He (boyfriend) got angered and even denied that he is responsible for the pregnancy.”*

*“He started saying that he was not responsible. I just told him if he thinks it belongs to someone else then it is okay” ………. The (her family members) asked me to abort it or else they would beat me up.”*

Pregnancy experience is often followed by fear, anxiety, and difficulty making decisions, even for prepared adult mothers. Early motherhood may be followed by rejection from the immediate family, especially the baby’s father (adolescent’s partner), which increases psychological vulnerability for this population. Pregnant adolescents face the added stress of adjusting to the new reality of parenthood without social and financial support from their families and significant others. Many of them experienced ridicule from relatives, friends, and neighbours around adolescent pregnancy and partner abandonment. Peer pressure, coercion, and seduction were identified as common causes of the first sexual experience, as shared by most participants.

*“No; I was obviously seduced.”*

*“He didn't give me alcohol, he just talked to me.”*

*“It is just wanting (willingness) because even that relationship started as a result of peer pressure; you want to be seen.”*

*“It is just the way you hear that others have done it (intercourse) and you also want to try it.”*

*“You just know life sometimes you don't have pads, you don't have whatever- books, whatever; so you get somebody who tells you "I will buy for you, that way.”*

*“It was just because of peer pressure.”*

*“That boy convinced me, and I couldn't resist because I was in love with him.”*

**Fig. 2:**
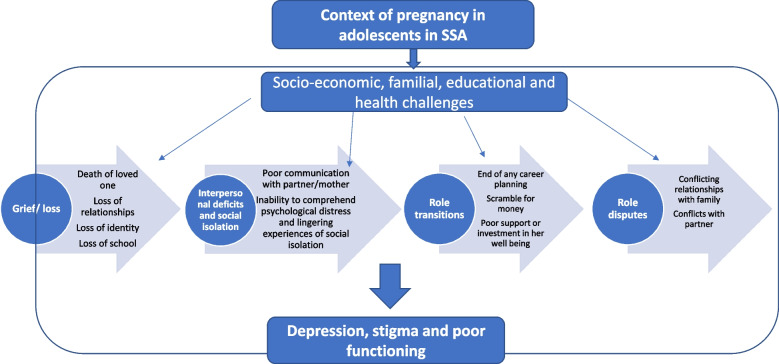
Interpersonal problems as a conceptual framework

The four problem areas of IPT, such as grief and loss, role transitions, interpersonal disputes/conflict, and loneliness /social isolation, did resonate in these interviews (see Figs. 1, 2, and Table 3).

Some participants had lost one of their parents, leading to experiences of unresolved grief. These adolescents voiced a great sense of neediness, poor self-esteem, and low capacity for assertiveness.

 “*Yes; mum passed on and I never found parental love.”*

*“You know we were raised by uncle, mum's brother. Mum died in two thousand and four.”*

Role transitions were especially dramatic for those in school when becoming pregnant and then experienced abrupt social changes as they transitioned to early motherhood. Pregnancy meant dropping out of school and being left to fend for themselves and their unborn children. In addition, pregnancy-related health difficulties like vomiting, persistent tiredness, headaches, and body aches could underscore the significant life change they were undergoing.

*“Used to go to school while pregnant but when my mother noticed that, I decided to move out (stop going to school)”*

*“The issues of girls not paying attention to the parents, running around with boys, so my sister took me and brought me here so that I study from here; I came here in the same year twenty seventeen then I again went to live with another sister of mine that is when I became pregnant. ”*

Interpersonal conflicts were reported as the interpersonal problem area most associated with early unanticipated adolescent pregnancy. Dropping out of school caused parents to be angry with the pregnant adolescent and led to misunderstandings within the family. Parents/guardians disagreed with the pregnant adolescents’ life choices and often expressed disappointment with their decisions. Male partners would also disown paternity and decline to take responsibility for the baby, thereby increasing tension, frustration, and interpersonal conflict for the pregnant adolescent.

*“He was worried because my parents could come anytime to pick me up……it was just the parents being frustrated …………….they quarrelled because I had not completed my studies.”*

*“He said that I should abort to show that the child is his, so I decided it is better that I forget him…I continued on my own…………It was not that easy because there are things that we went through; sometimes mum doesn't talk to you because she is also disappointed.”*

*“They became angry because they never thought that I was in grade 12, they thought that I am in form two (grade 10); so upon realizing that I was in form four (grade 12) they got angry.”*

*“They wanted me to go back home so that they can take me for a course (vocational training), but I refused.”*

## Discussion

### Overall findings and interview experience

Depression in pregnant adolescents living in impoverished settings in Kenya is a multidimensional phenomenon and a product and consequence of poor relationships and lack of timely, adequate social support. Among pregnant adolescents, the ratings for moderate-severe depressive symptoms were higher than the prenatal adult women [28].

This qualitative study with peripartum adolescents confirms that the framework of IPT problem areas helped us understand the sources of our participants' distress, suggesting that the use of IPT in this population is likely a good match for presenting needs, and offers critical skillset needed to improve communication, understanding of self and others, including the baby and in turn to be better understood by others. We have documented this in previous studies where similar needs were articulated [1, 3].

### Interpersonal problems as a framework to understand pregnant adolescents: appraising peers, caregiver and partner influences

Cultural psychology places the conceptualization of the self (known as “self-construal”) as central to understanding cultural differences in behaviors, thoughts, and emotions [29]. In our work around pregnant and parenting adolescents in Kenyan informal settlements, we delve into Kenyan cultural realities and how lived experiences and relational problems can be better understood against the early and unanticipated pregnancy. Several studies using IPT in Africa have pointed to the benefits of using these constructs to frame discourse around maternal health and well-being. These have at times been more valuable to engage households and communities in understanding the need for improved mental health. For example, studies on IPT in Uganda underscore the support such interventions add to community mental health, strengthening resilience to combat poverty and socioeconomic adversities [30] (see Fig. 1).

Figure 2 provides a schematic representation of putative interpersonal factors driving high rates of depression and impairment associated with adolescent pregnancy in Sub-Saharan Africa. We have used an enlarged definitions of grief and loss to include psychological losses as well as interpersonal deficits and social isolation problem areas basing it on our experience with young adolescents here. On grief and loss, we have added multiple losses such as loss of education, identity, relationships alongside death and bereavement. We have included within interpersonal deficits the construct of social isolation emanating from poor support networks and constant feeling of loneliness and isolation from their lived environment. In this regard, we do make some deviations from the classical model of IPT.

The source of psychological distress is the unintended and unplanned pregnancy which triggers and aggravates a range of adverse social determinants of health such as poverty, disengagement from school and education, poor health care access, and poor family support and well-being. In such an environment, there are cultural factors to consider in regulating emotions and how one experiences psychological distress. Our conceptual framework offers an interpersonal theory of depression, bringing out interpersonal psychotherapy's problem areas as crucial reference points for understanding the mental health of peripartum adolescents in the Kenyan context.

### Our study findings using the IPT framework

The experiences of our adolescent interviewees maps naturally onto the four IPT problem areas (grief, role disputes, role transitions, and interpersonal deficits). Several participants endorsed the loss of a parent or close family member (*loss and grief*) at an early age for adolescents, a predisposing factor for depression. IPT can address grief by facilitating the mourning process and helping the participant reestablish interests and relationships that can help fill some of the gaps left by the deceased [31]. Role disputes were commonly encountered in our sample: peer influence was one of the key reasons behind the first sexual encounter, leading often to the unwanted pregnancy and poor relationships with the boyfriend*.* Frequent reports of ACEs also exposed the participants to transactional sex in exchange for basic needs like sanitary pads and schooling items. Role disputes related to pregnancy, included perceived rejection by the family, ridicule by their peers, and male partners disowning responsibility for the pregnancy. Poor interpersonal relationships among youths and their parents have been reported to be associated with depression [32, 33] and commonly occurred in this sample. Role transitions were commonly reported. The pregnancy itself was defined as a role transition with resultant changes in relationships, finances, and living situation. A related transition commonly described was dropping out of school due to unintended and unplanned pregnancy. When the IPT definition of deficits was expanded to include current social isolation, numerous participants endorsed feeling alone and without social supports.

### Implications and future directions: extending IPT as an evidence-based intervention in this population

Originally, IPT was conceived as time-limited psychotherapy that focuses on interpersonal problems and crises that trigger and maintain psychological distress. First developed in the 1970s, IPT was initially provided to treat acute depression by Gerald Klerman and Myrna Weissman. They based it on Harry Stack Sullivan's, Adolf Meyer's, and others' work [34]. IPT posits that depression occurs in an interpersonal context and that helping the client resolve the interpersonal issues which trigger and maintain depression will dissolve the depressive episode. Furthermore, it hypothesizes that IPT decreases interpersonal distress and enhances social support by improving interpersonal skills building, and emotional processing [35].

In 2019, WHO launched group IPT to provide LMICs with a version of IPT that would be task-shifted to non-specialist workers given limited access to professional mental health providers in these countries. In Uganda, IPT-G was associated with improved school attendance and reduced conflict in families [30, 36].

IPT is now being used extensively for underserved clinical populations in LMICs [17, 37–41], suggesting that this is also a promising approach for pregnant adolescents in Kenya.

### Long-term implications of using this framework to understand vulnerable populations

Although IPT is an evidence-based treatment for depression, individual interventions are likely to have limited impact on broader social problems. Specifically, governments are accountable to their population and are therefore responsible for enacting systemic changes needed to improve collective wellbeing. In thinking about peripartum adolescents, intergenerational cycles of trauma, poverty, and poor attachment- from adolescent girls to their infants- must be addressed comprehensively. Interventions are needed at the level of mental health promotion, prevention of illness- both mental and physical, and providing timely access to care when an adolescent is at high risk for mental or physical illness. Without mental health becoming integrated into a broader system of program, these root causes cannot be addressed.

**Fig. 3:**
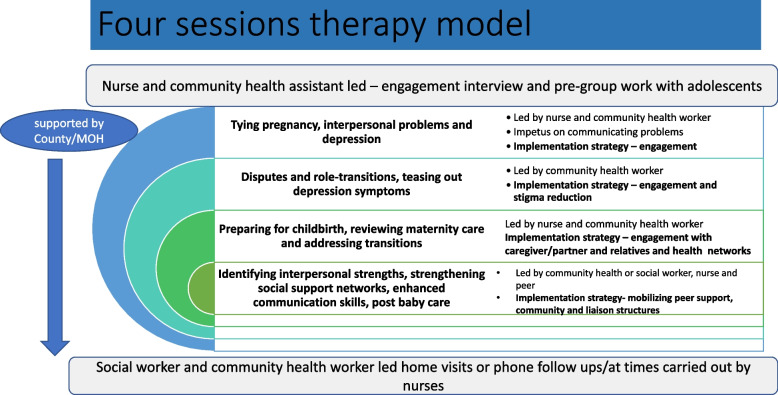
Proposal for modified intervention

Table 4 outlines a series of barriers and facilitators to delivering IPT that have emerged through this qualitative work and Fig. 3 provides proposed modifications of IPT-G to better meet the needs of depressed, pregnant adolescents. These recommended strategies include focusing on their everyday relationship challenges and psychosocial problems, developing strategies to address role transitions and disputes in key familial and romantic relationships and sharing coping strategies to address social isolation, loneliness and grief.

**Table 4:** Barriers and opportunities for IPT contextual adaptation and implementation

**Barriers to depression intervention development**
*Intervention specific***-**mhGAP intervention guide needs structured MH system strengthening strategies beforehand, Group IPT is acceptable but needs modifications in format and dose, cost of adaptation and training are considerable, training requires a sustainable supervisory structure
*Adolescent specific*- Pregnant adolescents have poor mental health service access, little family or community support, poor access to educational or vocational, parenting training, often without male partner support
**Opportunities for using IPT for depression intervention**
*Intervention specific*- National level ratification & proposed scale up of WHO mhGAP-IG. High acceptability of G-IPT, demonstrates feasibility delivered in health facilities by lay health workers, improves depression and social support in peripartum adolescents System specific- Politically receptive to SRH, adolescent centered services and integrating MH, peer models championed
*Adolescent specific*- focusing on their everyday relationship challenges and psychosocial problems, developing strategies to address role transitions and disputes in key familial and romantic relationships and sharing coping strategies to address social isolation, loneliness and grief

Table showing barriers and opportunities for contextual adaptation and implementation of interpersonal psychotherapy

### Limitations

While this study provides an understanding of interpersonal challenges facing peripartum adolescents, there are some limitations. We noted that pregnant adolescents needed a lot of encouragement to explore issues around mental health and that we needed to probe these issues with follow-up questions. We felt that we needed to provide requests for clarifications and additional prompts as the interviews progressed. Although 23 participants is a relatively large sample for a qualitative study, it cannot capture the voices and concerns of all depressed, pregnant teenagers. However, authentically knowing their perspectives and voices is essential to understanding their challenges.

## Conclusion

Pregnant adolescents experience multilayered socio-emotional problems. These interpersonal problems are a precursor to and consequence of poor mental and physical health triggered by early unanticipated pregnancy. Our work has demonstrated the significance of the interpersonal problem areas in the lives of these vulnerable adolescents. In addition, we have tried to make a case for the use of IPT’s interpersonal problem areas as a conduit for effective intervention.

## Data Availability

The datasets generated and/or analyzed during the current study are not publicly available due but are available from the corresponding author on reasonable request.
